# Preimplantation Genetic Diagnosis: Prenatal Testing for Embryos Finally Achieving Its Potential

**DOI:** 10.3390/jcm3010280

**Published:** 2014-03-17

**Authors:** Harvey J. Stern

**Affiliations:** 1 Division of Reproductive Genetics, Genetics & IVF Institute, 3015 Williams Drive, Fairfax, VA 22031, USA; E-Mail: hstern@givf.com; Tel.: +1-703-698-7355; Fax: +1-703-698-1137; 2 Departments of Obstetrics and Gynecology, Pediatrics and Human Genetics, Virginia Commonwealth University, Richmond, VA 23298, USA

**Keywords:** preimplantation genetic diagnosis, chromosomal microarray, embryo biopsy, next generation sequencing, inherited genetic disorders

## Abstract

Preimplantation genetic diagnosis was developed nearly a quarter-century ago as an alternative form of prenatal diagnosis that is carried out on embryos. Initially offered for diagnosis in couples at-risk for single gene genetic disorders, such as cystic fibrosis, spinal muscular atrophy and Huntington disease, preimplantation genetic diagnosis (PGD) has most frequently been employed in assisted reproduction for detection of chromosome aneuploidy from advancing maternal age or structural chromosome rearrangements. Major improvements have been seen in PGD analysis with movement away from older, less effective technologies, such as fluorescence *in situ* hybridization (FISH), to newer molecular tools, such as DNA microarrays and next generation sequencing. Improved results have also started to be seen with decreasing use of Day 3 blastomere biopsy in favor of polar body or Day 5 trophectoderm biopsy. Discussions regarding the scientific, ethical, legal and social issues surrounding the use of sequence data from embryo biopsy have begun and must continue to avoid concern regarding eugenic or inappropriate use of this technology.

## 1. Introduction

Preimplantation genetic diagnosis (PGD) is a form of prenatal diagnosis that is performed on early embryos created by *in vitro* fertilization (IVF). In comparison to other established methods of prenatal diagnosis, such as chorionic villus sampling and amniocentesis, PGD is not performed on an ongoing intrauterine pregnancy in the late first or early second trimester, but on embryos developing in the IVF laboratory prior to transfer to the uterus. Despite some misconception to the contrary, PGD is not a therapeutic procedure for embryos; there are no changes to the DNA or any other genetic-related structures. It is solely a diagnostic procedure that can identify whether a specific embryo carries a single gene disorder for which the couple is at-risk or a chromosome abnormality that could lead to either failed implantation, subsequent miscarriage or the birth of a child with physical and/or developmental disability. This information is used by the couple and their physicians to make decisions on which embryo(s) should be transferred to the uterus and will with high likelihood result in a normal pregnancy. Since multiple embryos are created in IVF, PGD has a distinct numerical advantage over testing of a single ongoing pregnancy. The greater the number of embryos created, the greater the chance that genetically normal embryos can be identified. The level of selection is that of choosing which embryos can be transferred in a fresh IVF cycle or cryopreserved for future use, *versus* those predicted to be affected with an abnormality. This is in contrast to chorionic villus sampling or amniocentesis, where a decision has to be made whether to terminate an ongoing affected pregnancy. For many couples, PGD is the more, or only, acceptable choice.

Early research in PGD dates back to Edwards and Gardner [1], who, in 1968, biopsied a rabbit blastocyst and performed Barr body analysis to determine gender. PGD became feasible in humans with the first successful IVF by Edwards and Steptoe in 1978 [2]. Other research on PGD was performed over the next 10 years on mouse blastomeres, including the demonstration by Monk and Handyside in 1988 of the feasibility of using PGD to detect a single gene disorder [3]. The first successful application of PGD in humans was by Handyside and colleagues in 1990 [4], who performed sexing of embryos by polymerase chain reaction (PCR) for the presence of the Y chromosome sequence to avoid males affected with X-linked adrenoleukodystrophy and X-linked mental retardation. Shortly thereafter, successful PGD was reported for cystic fibrosis [5], α-1 antitrypsin deficiency [6] and many other single gene disorders.

The other area where PGD has played a major role in assisted reproduction is for the detection of chromosome abnormalities in embryos due to inherited structural rearrangements, such as translocations, inversions or duplications/deletions, as well as age-related aneuploidy, with the goal of improving the outcome of IVF. This was made possible by the use of fluorescence *in situ* hybridization (FISH) in biopsied blastomeres by Griffin [7] and Griffo [8], who initially used probes for the X and Y chromosome to determine gender, and later by Munne [9], who developed protocols for multicolor-FISH to screen up to five chromosomes simultaneously. FISH has, for the most part, been replaced by molecular technologies for aneuploidy testing, including array comparative genomic hybridization (aCGH), single nucleotide polymorphism (SNP) arrays, quantitative PCR (qPCR) and, most recently, next generation sequencing (NGS)-based protocols. The potential applications of PGD have expanded dramatically with the easy availability of sequence-based information on pathologic sequence changes and copy number variations, along with the remarkable ability of current sequencing platforms to rapidly generate large amounts of sequence data.

In this review, I will trace the development of PGD from a boutique technique performed in only a few centers worldwide, to an established prenatal diagnostic procedure practiced, at least in some form, by the majority of IVF centers. The changes that have occurred in the indications for PGD and the type/timing of embryo biopsy will be discussed along with the introduction of advanced DNA analytic technology, including comparative genomic hybridization (CGH) and next generation sequencing (NGS). Finally, we can only start to predict what the future holds for PGD; however, it is clear that discussion is needed to more carefully focus on the ultimate goal(s) of PGD, or the technology will be viewed skeptically as a precursor to eugenics and genetic engineering of children to produce “designer babies”.

## 2. Utilization of PGD

The actual number of PGD cycles that have been performed to date can only be estimated. In the U.S., until recently, there was no requirement (nor mechanism) to report the use of PGD in the clinical data submitted to the Society for Assisted Reproductive Technology (SART), which publishes a database of IVF cycles and outcomes for U.S. clinics. Two international working groups for PGD have been formed with the goal of collecting and mining data on PGD cycles performed and to act as a forum for participating PGD centers to exchange information, develop quality control measures and best practice standards, as well as to provide educational opportunities and hands-on workshops. The ESHRE PGD Consortium was established in 1997 and has collected data each year thereafter. At the 2013 ESHRE conference, the most recent data from 115 registered centers was reported [10]. The dataset contained information on 51,589 PGD cycles with the breakdown of indications shown in Table 1.

**Table 1:** Data from the ESHRE Preimplantation Genetic Diagnosis (PGD) Consortium, including their 14th data collection (Traeger-Synodinos *et al.* 2013 [10]) and the recorded indications for PGD in 51,589 cycles.

Indication	Cycles	Percentage
Single gene disorders	11,084	21%
Aneuploidy screening	30,033	58%
Inherited chromosome abnormalities	8104	16%
Sexing for X-linked disease	1603	3%
Non-medical (social) sexing	765	2%

In addition to the ESHRE PGD Consortium, Verlinsky and Kuliev in Chicago had organized an International Working Group on PGD, which held meetings dedicated to education and advancement of the field of PGD. In 2003, this group became the PGD International Society (PGDIS) and held its first meeting in 2005. On their website (www.pgdis.org), the organization currently estimates that approximately 100,000 PGD cycles have been performed worldwide over the past 23 years. Nearly 80% of these cycles have been performed for aneuploidy screening, 12% for single gene disorders, 6% for chromosome rearrangements and 2% for sibling human leukocyte antigen (HLA) matching. Both this and the ESHRE surveys (which likely contain overlapping data) confirm that aneuploidy testing is the major indication for PGD. This is, however, performed more frequently in the United States as opposed to Europe, where this indication had decreased for the previous seven years, due to concerns regarding the accuracy of FISH-based chromosomal analysis and the lack of data showing an increase in the delivery rate in IVF cycles where FISH-PGD was used as compared to matched IVF cycles where PGD was not performed.

A survey from the Johns Hopkins Public Policy Center developed by Baruch *et al.* [11] was mailed to 415 ART clinics in the Unites States in 2008 to determine how many clinics utilized PGD for various indications. Of the 137 responders who offered PGD services, the indications are shown in Table 2.

**Table 2:** Results of a survey of 137 *in vitro* fertilization (IVF) centers regarding the indications for which PGD was offered in their clinic. PGD for aneuploidy testing, genetic disorders and structural chromosome rearrangements was performed by the majority of the respondents [11].

Indication for PGD	Percent of Clinics Offering This Type of PGD
Aneuploidy testing	93%
Single gene disorders	82%
Structural chromosome rearrangements	67%
Fetal sex for X-linked disease	58%
Non-medical sex selection	42%
Avoid adult onset disorder	28%
HLA * typing with disease testing	24%
HLA typing without disease testing	6%
Selection for a disability	3%

* HLA = human leukocyte antigen.

Of the 186 clinics that responded to the survey, 74% indicated that they have provided PGD services. All clinics performing >500 cycles per year reported that they have offered PGD. The single most common indication was using PGD for aneuploidy testing to attempt to improve the outcome of IVF or for couples with recurrent pregnancy loss or failed IVF. Eighty-two percent of clinics indicated that they perform PGD for couples at-risk for autosomal single gene disorders, such as cystic fibrosis, Tay Sachs disease, sickle cell disease and Myotonic dystrophy. Single gene disorders comprised 12% of all PGD cycles in the U.S. Twenty-eight percent of clinics offered IVF/PGD to avoid adult onset disorders, such as Huntington disease, hereditary breast and ovarian cancer and Alzheimer disease. Using PGD to screen embryos for diseases that will not develop until adulthood or for mutations that confer a heightened risk (as opposed to a certainty) for developing a particular disease raises issues of how to weigh the possible benefits of PGD to the future child and adult against the known and unknown risks of PGD and IVF. Having a genetic mutation associated with a particular disease, such as hereditary breast cancer, does not mean there is a certainty that the disease would develop, and children with adult onset disorders could expect to remain healthy for decades before symptoms, if any, would begin.

Some couples have used PGD to attempt to have a baby who is an immunological match for an existing seriously ill child, where the baby’s cord blood is then used for stem cell transplantation. This use of PGD is known as human leukocyte antigen (HLA) typing. This type of PGD was first reported by Verlinsky [12,13], and overall, 23% of IVF clinics have performed PGD for HLA typing in conjunction with genetic analysis to ensure the baby will also be free of the genetic disease affecting the older sibling. Some families have sought HLA typing to have a baby who is an HLA match for an older child when the disease is not inherited and for which the future baby is not at risk. Six percent of IVF clinics have provided PGD in such cases. Conceiving a child for the purpose of curing an older sibling is controversial; when one child has been selected to serve as an immunological match for another, the term “savior baby” is frequently used. It has been argued that in cases where the disease affecting the older sibling has no hereditary basis, any potential risks from IVF or embryo biopsy would be imposed upon the younger child without any benefit to that child [14].

PGD can be used to select the sex of an embryo, either to avoid a genetic disease in males caused by a mutation on the X chromosome (X-linked disease) or simply to satisfy the gender preference of the future parents. When PGD for sex selection is done in the absence of medical indications, it is often referred to as “non-medical sex selection” or “social sexing”. According to the survey, 42% of IVF clinics have provided PGD for non-medical sex selection. Of all PGD cycles reported in 2005, non-medical sex selection was performed in 9% of cases [15]. Nearly one-half of the clinics reported that they will provide gender selection under any circumstances, while others will only provide “family balancing” for a second or subsequent child of the under-represented sex already present in the family. The use of PGD for sex selection has triggered ethical concerns that sex selection amounts to sex discrimination. It is also assumed that most couples would desire a male child. While this may be true for cultural reasons in some ethnic groups, most studies in the U.S. have shown that there is a nearly equal desire for females as for males among couples wishing to select the gender of their child through PGD [16] or in cases of fetal reduction [17]. Non-medical sex selection is prohibited in Europe, China and Australia, although since sex chromosome information is reported in aneuploidy testing, non-reported cases are likely to occur.

An uncommon use of PGD involves the selection of an embryo for the presence of a particular disease or disability, such as deafness or dwarfism, in order that a child would share that characteristic with his or her parents. Only four IVF clinics in the Baruch *et al.* 2008 survey [11] indicated that they have provided this service. Of all the controversial uses of PGD, this one occurs least often, but nevertheless attracts significant public attention. Most IVF clinicians have indicated that they will not provide PGD to families who seek to select for a disability, since in their mind, the goal of PGD is to help produce healthy children.

Utilization of PGD services is regulated or prohibited in many countries based on national and/or local laws [18]. The majority of countries require that PGD be limited to conditions that produce significant, incurable medical illness and in which there is a significant risk that the fetus will be affected with the condition through typical Mendelian inheritance. In France, India, the Netherlands and the U.K., access to PGD is regulated by local law and allowed only with a specific license. Austria, Switzerland and Western Australia prohibit PGD. In Germany, only as many embryos may be generated *in vitro*, as will be transferred in the same IVF cycle (up to a maximum of three), and since 2011, analysis of the first polar body can be used to select oocytes for fertilization. PGD for serious genetic disorders has also recently been allowed. Switzerland has among the most restrictive legislation in the world on medically-assisted reproduction, and the development of more than three embryos outside the mother’s body is prohibited. Italy’s IVF regulations passed in 2004 require that all fertilized embryos be transferred, and therefore, PGD was limited only to analysis of the first polar body prior to fertilization. In 2009, PGD for single gene disorders and translocations was allowed after an Italian Supreme Court decision that the restrictions were unconstitutional [19]. For a further discussion of European regulation of PGD, see Corveleyn, *et al.* [20,21].

## 3. Tissue Biopsy for PGD

Decisions regarding the optimal time to perform PGD biopsy and testing involve careful consideration of multiple factors, including: (1) When in embryologic development is the abnormality first identifiable? For example, mitotic nondisjunction will be missed in polar body analysis; (2) Are genetic changes in the specimen indicative of abnormalities in the embryo as a whole? Chromosome mosaicism in the blastomere and the capacity of the embryo for self-correction are key questions in this situation; (3) Does the timing of the biopsy relative to transfer allow sufficient time for the analysis? (4) Does the biopsy itself compromise the ability of the embryo to develop normally [22]?

PGD may be performed at three different embryonic developmental stages. The first involves biopsy of the polar bodies just prior to conception (first polar body) and after fertilization (second polar body). Day 3 cleavage cell biopsy involves blastomere removal at the 5–8 cell embryonic stage. Finally, trophectoderm or blastocyst biopsy is performed on Day 5–6 embryos that consist of approximately 120 cells. Five to eight cells are removed from the trophectoderm as it “hatches” through the zona pellucida.

### 3.1. Polar Body Biopsy

The first and second polar bodies are by-products of the meiosis of the egg, and their removal is believed to be less harmful to the embryo than either blastomere or trophectoderm biopsy, which, by their nature, reduce the embryonic cell number [23]. Polar body biopsy is performed early in the IVF process, which can allow extra time for PGD testing to be performed prior to the fresh transfer. The basis of polar body screening is that any abnormality identified is associated with a corresponding error in the oocytes. For example, if the polar body shows two copies of chromosome 21, the corresponding oocytes will have no copies, and an embryo will be monosomic for chromosome 21. For women who carry recessive single gene mutations or are affected with a dominant genetic disorder, if the mutation is present in the polar body DNA, it is assumed that the egg carries the normal allele and, thus, will lead to a normal embryo [24].

The first polar body is present before fertilization, and therefore, with its biopsy, one can obtain pre-conceptual information on the egg, which can then guide clinicians as to which oocytes should undergo fertilization [6]. This is particularly useful where limited numbers of embryos can be created due to local regulations. The limitations of this approach are that no information on the paternal DNA contribution can be deduced, nor will mitotic nondisjunction be detected. Especially for embryonic chromosome anomalies, the first and second polar body need to be evaluated, which doubles the cost of the analysis. Even in this case, the interpretations of the results from polar body analysis are not entirely straightforward. This is because not all errors in the oocyte occur from nondisjunction in the first phase of meiosis [25]. Premature separation of sister chromatids in Meiosis I has been shown to be a significant cause of aneuploidy in oocytes and complicates the interpretation of polar biopsy results [22,26,27]. The polar body biopsy process requires entering the perivitelline space through an opening in the zona pellucida produced by either laser or mechanical dissection. This latter method was perfected by Yuri Verlinsky of the Chicago Reproductive Genetics Institute, who used two perpendicular cuts in the zona to create a flap to introduce a biopsy pipette. The two polar bodies can be obtained at the same time or sequentially. The optimal time for biopsy seems to be 6–9 h post fertilization [28]. After that time, the first polar body has a tendency to degenerate, compromising FISH or DNA-based testing.

Due to the limitations on PGD in Europe described above and because of the frequent finding of chromosomal mosaicism in cleavage stage embryos, an ESHRE-sponsored clinical trial in Europe was undertaken to access whether polar body diagnosis by array CGH provided a reliable, accurate and timely methodology to determine the maternal component of the chromosomal status of the embryo [29]. This showed that polar body-based PGD provided acceptable accuracy, but still greater than 10% of normal embryos could be given an incorrect diagnosis of aneuploidy [30].

### 3.2. Cleavage Stage (Blastomere) Biopsy

Cleavage stage biopsy is the most widely used biopsy technique for PGD and involves the removal of a single embryonic cell on Day 3 of development (6–8 cell stage) after opening of the zona pellucida with either laser, mechanical dissection or exposure to acidic Tyrode’s solution. The blastomere is removed after introduction of the biopsy pipette by aspiration or by extrusion of the cell with pressure on the outside of the zona [31]. The embryos just prior to biopsy are transferred to calcium/magnesium-free media to aid in blastomere removal [32]. Some centers removed two blastomeres, until this was shown to produce a greater decline in live birth than a single-cell biopsy [33,34]. Cleavage-stage biopsy, unlike PB biopsy, is able to detect maternally and paternally-derived chromosome defects, as well as some mitotic defects. Biopsy on Day 3 embryos allows 2–3 days for PGD analysis to be completed if a fresh embryo transfer is desired.

The presence of embryo mosaicism is a major limiting factor in the interpretation of PGD results in cleavage-stage embryos. Mosaicism affects 15%–80% of embryos at Day 3, and significant mosaicism remains present at the Day 5 blastocyst stage [35,36,37]. This raises the question as to whether a biopsied blastomere is an accurate representation of the embryo as a whole. In addition, it appears that the ability of the early embryo to undergo self-correction by either selective apoptosis or allocation of abnormal cells to the trophectoderm is limited [38].

The consideration of cleavage stage blastomere biopsy must take into account the impact of the biopsy on embryo progression, implantation and development. Most investigators were initially encouraged by the early results of Hardy [39], which suggested that blastomere biopsy did not inhibit the ability of embryos to progress to the blastocyst stage. This data, however, was based on good quality embryos and likely does not accurately represent the spectrum of embryo quality seen in a typical IVF cycle. Cohen *et al.* [34] pointed out that based on a blastomere cryopreservation survival model developed by Edgar [40], the biopsy of a single cell at the eight-cell stage would lead to a decrease in implantation of 12.5%, and a two-cell blastomere biopsy would produce a 25% decrease in implantation. This coupled with the significant incidence of mosaicism in cleavage stage embryos, as well as the high incidence of allele dropout (ADO, see below) led to the interest in obtaining a multicellular trophectoderm biopsy of the Day 5 blastocyst as an alternative to single-cell blastomere biopsy for use in PGD.

### 3.3. Blastocyst (Trophectoderm) Biopsy

The human blastocyst contains approximately 130 cells distributed between the inner cell mass, which will develop into the fetus proper, and the surrounding trophectoderm cells, which will become the placenta and fetal membranes. Clinical use of blastocyst biopsy was initially reported by McArthur and the Sydney IVF group [41,42] and entails the biopsy of 5–10 trophectoderm cells on Day 5 or 6 after laser assisted hatching on Day 3, which creates a 25–30 μm opening in the zona pellucida, allowing herniation of the trophectoderm cells through the zona. The cells are stretched out in a biopsy pipette and removed with a laser. Since trophectoderm cells are removed and cells from the inner cell mass are avoided, it is believed that blastocyst biopsy causes less harm to the embryo than Day 3 (blastomere) biopsy. Trophectoderm biopsy has the advantage of collecting multiple cells, which can mitigate the effects of mosaicism and ADO when genetic analysis is performed by PCR. In addition, since only about 50% of fertilized embryos will progress to blastocysts, fewer embryos are biopsied compared to Day 3 blastomere or polar body biopsy. This can also be a disadvantage, as laboratories performing blastocyst biopsy must be capable of embryo culture to Day 5 or 6. Trophectoderm biopsy will also result in fewer embryos being available for transfer, since compacting embryos and morulas, which can occasionally produce pregnancies with Day 5 transfers, are generally excluded from analysis.

Scott *et al.* [43] have provided evidence for the superiority of blastocyst biopsy in a randomized clinical trial using a paired design where each patient acted as their own control group. In this study, couples undergoing IVF had two embryos selected from the same treatment cycle for transfer on either Day 3 or Day 5. Prior to the embryo transfer, one of the two chosen embryos randomly underwent biopsy, either on Day 3 (blastomere) or Day 5 (trophectoderm). The biopsied material did not undergo aneuploidy testing, but instead, DNA fingerprinting was performed, so that it could be determined which embryo implanted in singleton conceptions. The DNA pattern of the delivered child was determined from prenatal testing or fetal DNA obtained from maternal serum and was correlated with either the implanted or non-implanted embryo. Evaluation of biopsy performed at the Day 3 cleavage stage (blastomere) showed that it resulted in a relative decrease in embryo implantation of 39%. For biopsies performed at the Day 5 (blastocyst) stage, there was no difference in implantation and delivery rates between biopsied and non-biopsied embryos. These results provide strong evidence that cleavage-stage biopsy is more detrimental to the embryo than biopsy at the blastocyst stage, and this leads to poorer clinical outcomes. It is likely that more PGD centers will adopt trophectoderm biopsy in the future. The most recent ESHRE dataset [10] indicated that only 2.3% of biopsies were performed on Day 5 or 6, as compared to 79.8% at the Day 3 cleavage-cell stage.

## 4. Technique of Genetic Analysis in PGD

### 4.1. Single Gene Disorders

PCR has been the preferred method of diagnosis in PGD for single gene disorders. Nearly all genetically inherited single gene disorders where sequence information on the mutation is known can be detected by PGD. Diagnostic protocols for over 200 different single gene disorders have been reported, with the most common being for cystic fibrosis, spinal muscular atrophy, hemoglobin disorders, such as β-thalassemia, Huntington disease, fragile X syndrome and myotonic dystrophy. As opposed to the analysis of DNA from blood or amniocentesis/CVS samples, PCR in PGD usually begins from very limited amounts of DNA found in blastomere or trophectoderm biopsies. Single cells contain approximately six picograms of DNA, and for many PCR tests, 250 nanograms or more of DNA is required as the starting material. This implies that roughly a 40,000-fold amplification in DNA concentration is needed. One problem that became apparent was that the large number of PCR cycles needed to generate a product led to an increasing rate of the introduction of errors in the DNA. This was overcome by the development of “nested” PCR by Holding and Monk [44]. This technique uses a first round of amplification with an outer set of primers, followed by a second round using a set of inner primers, which when using the first round product as a template, amplifies a smaller PCR product inside the outer primers, which is robustly produced with high accuracy.

The low initial starting concentration of DNA in embryo samples predisposes PCR to several other complicating factors, such as contamination with external DNA and allele dropout (ADO). Contaminating DNA can originate from the technician performing the PCR, from carryover of other PCR products or controls from earlier amplifications or from cumulus cells or sperm adherent to the zona. Careful cleaning of the embryo prior to fertilization and the use of intra-cytoplasmic sperm injection (ISCI) of a single sperm for oocyte fertilization can avoid this latter type of contamination. Operators typically dress in surgical gowns and perform the PCRs in laminar flow hoods situated in “clean rooms” to limit external DNA contamination.

Allele drop out (ADO) refers to preferential amplification or failure of the amplification of one of the alleles present in a heterozygous sample. This could lead to misdiagnosis of a heterozygous embryo as a homozygous affected sample, in which case, a normal embryo would be excluded from the cohort of embryos available for transfer. In the case of a dominant genetic disease, ADO can cause an abnormal embryo to be misdiagnosed and transferred in error as “normal”. ADO can occur if the annealing of one of the two PCR primers is less efficient. Alternatively, inefficient cell lysis or degenerated DNA can theoretically lead to ADO. The frequency of ADO is variable and, in PCR of single blastomeres, can be seen in 5% to over 20% of amplifications [45,46].

The early evaluation of PCR products included the analysis of products of radioactive-labelled primers or nucleotides, as well as ethidium bromide staining of DNA in agarose or polyacrylamide gels after restriction enzyme digestion. These methods have been replaced by fluorescent labeled probes or nucleotides, yielding products detected and sized by capillary electrophoresis. The most frequently utilized method for mutation detection is DNA primer-extension minisequencing [46] in which a probe is developed that extends up one base prior to the first nucleotide of the gene alteration. Single base-pair changes or small deletions/duplications can then be detected by the extension of the primer with fluorescent-labeled dideoxynucleotides, which will identify the next base in the target sequence and then stop, due to the chain-termination effect of dideoxynucleotides. Commercially available kits, such as the ABI SNapShot™ protocol (Life Technologies, Carlsbad, CA, USA), can be used for this purpose. Deletions and duplications can also be detected by that sizing of a PCR product, including the region of interest. The PCR reactions include primers for multiple target sites, not only for the area containing the mutation under consideration, but for closely linked polymorphic short tandem repeat (STR) markers, which can be used to detect DNA contamination and ADO [46]. This multiplex fluorescent PCR protocol led to a significant decrease in the number of analyses compromised by contamination or ADO.

**Figure 1:**
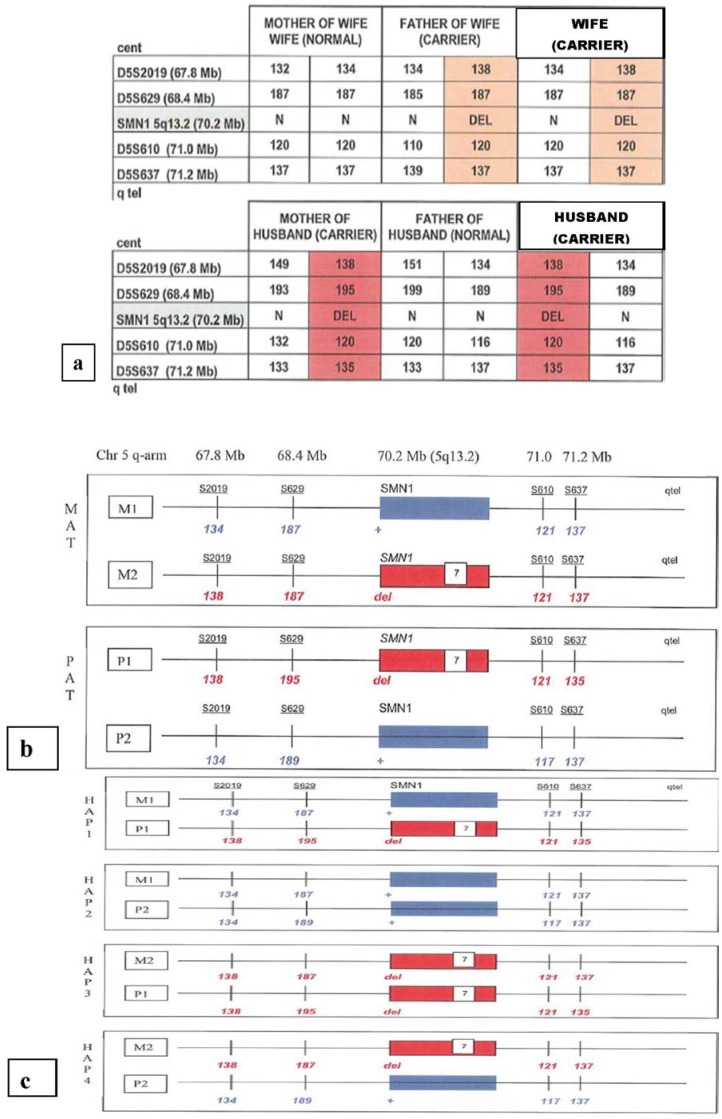
Pre-PGD workup for a family with a previous child with spinal muscular atrophy. Panel (**a**) shows how the study of both parents and grandparents allows the phasing of the *SMN* mutation relative to polymorphic short tandem repeat (STR) markers; panel (**b**) shows the maternal and paternal haplotypes M1, M2, P1 and P2 and the distance of the STR markers from the *SMN* gene; panel (**c**) shows the four predicted fetal haplotypes. These reflect a Hardy–Weinberg equilibrium of one homozygous non-carrier, two heterozygous carriers and one that is homozygous and affected. Short tandem repeat markers linked with the *SMN* mutation are shown in red. DEL indicates the presense of the exon 7 (840 C>T) mutation.

A pretest work-up typically involves obtaining DNA samples from the at-risk couple, as well as an affected child or grandparents to allow phasing of the STR haplotype with the mutation being tested. This allows the determination of the two maternal and two paternal haplotypes, as shown in Figure 1. This analysis aids in the interpretation of the results of PGD testing, although recombination of STR markers with the genetic mutation can be seen.

Newer PCR protocols have been developed using whole genome amplification (WGA) as a universal first step after biopsy of a single blastomere or 5–9 cells from a trophectoderm biopsy. This allows the analysis of a number of different sequences, such as compound heterozygous mutations of the *CFTR* gene, as well as allowing parallel testing of single gene disorders and linked STR markers or aneuploidy testing by aCGH. There are a number of methods for WGA that have been developed. Early protocols included primer extension PCR [47], degenerative oligonucleotide primed (DOP) PCR [48] or blunt end ligation and PCR amplification (GenomePlex™, Rubicon Genomics, Ann Arbor, MI, USA) [49]. All of these methods were dependent on PCR and carried the potential for uneven representation of the genome by preferential amplification or ADO. Multiple displacement amplification (MDA) is a WGA technology based on isothermal “rolling amplification” after the binding of random hexamers to the target sequence [50,51]. Despite the isothermal amplification in MDA protocols, ADO rates have been found to be at least 4% with SNPs [51], while others found a much higher ADO rate closer to 25% for linked STR markers used for PGD haplotyping [52,53]. The significant issues with ADO in single gene disorder PGD can be alleviated by the inclusion of multiple cells in the biopsy sample, which is why many centers are now moving toward trophectoderm biopsy, where 5–10 cells are typically obtained. In our lab, using trophectoderm biopsy and GenomePlex™ amplification, we have encountered an ADO rate of approximately 5% in single gene disorder PGD. Zong *et al.* [54] have reported on a new WGA method of multiple annealing and looping-based amplification (MALBAC), which is reported to significantly reduce amplification bias and provide 85%–95% genome coverage with 25X sequencing depth compared to 72% coverage at the same sequencing depth for the MDA protocol.

The GenomePlex™ system presents another issue, in that the average size of the amplified product is 500–1000 bp. In using this DNA for single gene disorder testing, we have found it important to limit the amplicon size, such that PCR products are smaller than the average WGA DNA product length and, therefore, contained in a single fragment. The use of larger amplicons leads to the failure of the PCR to include the desired sequence. Tran *et al.* [55] showed that this approach is successful in developing PGD tests and analyzing results for different classes of mutations, including single base pair changes, deletions/duplications and triplet repeat expansion mutations along with STR repeats, all with a low ADO rate.

It is likely that all WGA protocols result in some biased amplification, leading to decreased genomic coverage. Newer products are now being developed to address this bias, as well as producing highly accurate and representative amplification. As interest in more extensive sequencing in PGD develops, these issues will become more critical; however, at the moment, more limited evaluation, such as single gene sequencing and shotgun sequencing-chromosome counting protocols, seems to produce acceptable results with some current WGA methods (see below).

### 4.2. Age-Related Chromosome Aneuploidy

The most common use of PGD involves the analysis of embryos for chromosome aneuploidy arising from either meiotic mal-segregation in oocytes or (less commonly) sperm or mitotic abnormalities of embryos after syngamy, which results in mosaicism; defined as the presence of two or more cytogenetically defined cell lines in the same embryo. Chromosome aneuploidy is strongly correlated with maternal age, however; mitotic abnormalities seem to be age-independent [56]. Embryos that are aneuploid often result in developmental arrest, failed implantation or subsequent miscarriage [57]. Chromosome analysis of miscarriage products by aCGH shows that approximately 70% of first trimester miscarriage is due to aneuploidy [58,59]. Since chromosome anomalies are the major cause of unsuccessful IVF or pregnancy loss, it was proposed that identification and preferential transfer of euploid embryos in IVF would lead to higher implantation and birth rates with a lower miscarriage rate [9,60]. This concept was felt to make perfect sense, and it was initially expected that aneuploidy testing would result in a major improvement in IVF outcome, especially in couples with recurrent pregnancy loss or recurrent IVF failure. Since standard metaphase karyotyping cannot be rapidly performed on polar bodies, blastomeres or trophectoderm cells, a different technology of interphase FISH was introduced to evaluate the chromosomal content of embryonic cells. Clinically, FISH was first performed for sexing, using X and Y probes to avoid the transfer of embryos with X-linked genetic disease. Shortly thereafter, more extensive chromosome testing was developed using protocols that score 5–12 chromosomes in multiple rounds of hybridization using centromeric or locus-specific FISH probes [7,8,9]. Testing was limited by the fact that there were only five visible spectrum fluorochromes available to label FISH probes, so testing needed to be performed in multiple sequential rounds of hybridization, stripping and re-hybridization; each round performed with decreasing efficiency.

This form of PGD, which did not involve genetic disease testing and was designed to improve the outcome of IVF, was termed preimplantation genetic screening (PGS). The term was first used in Europe, apparently to distinguish chromosome testing from single gene disorder analysis, which was considered a less controversial use of PGD. The name PGS is a misnomer, as aneuploidy testing by PGD is clearly not a screening test in that clinical action (*i.e.*, the non-transfer of embryos diagnosed as abnormal) is taken based on the results of the testing. This is in contrast with the classical definition of screening tests, which alters one’s odds of having a specific condition without providing a definitive, actionable diagnosis. An example of a screening test is first trimester combined screening (ultrasound and blood analyte measurement), which does not provide definitive chromosomal analysis, but adjusts a patient’s prior risk based on maternal age alone.

**Figure 2:**
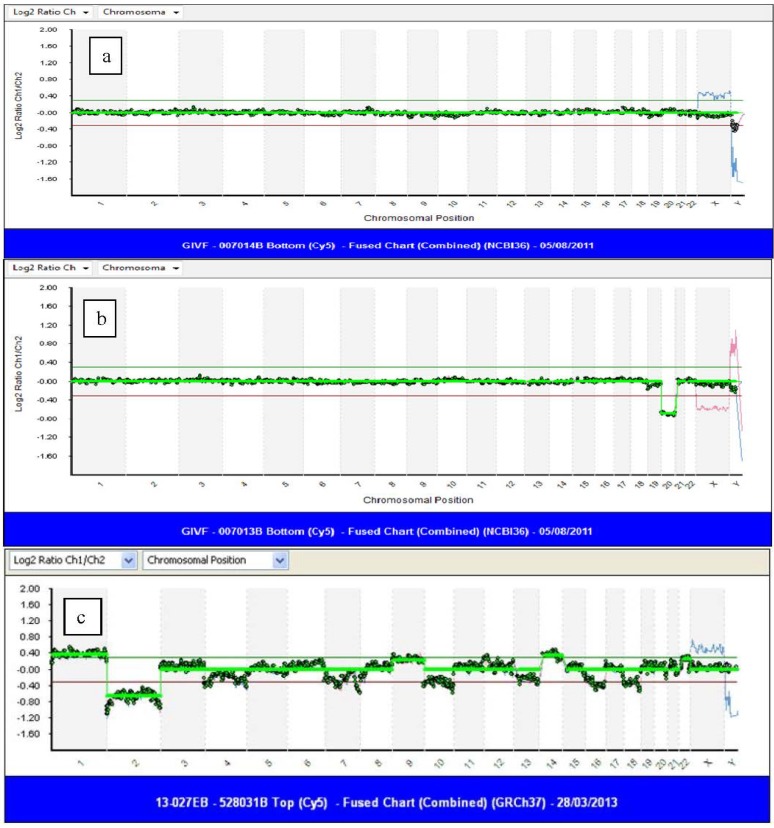
Array comparative genomic hybridization (aCGH) tracing after trophectoderm biopsy: (**a**) normal male embryo (female embryo control in blue); (**b**) female embryo with monosomy for chromosome 20 (male control in red); (**c**) an excellent quality blastocyst showing chaotic chromosome abnormalities. Nearly every chromosome is aneuploid.

Most chromosome studies performed prior to 2007 on cleavage stage embryo biopsy samples utilized FISH for analysis and showed significant levels of mosaicism throughout preimplantation development [36,61]. A systematic review of chromosomal mosaicism in preimplantation embryos, where every cell of an un-transferred embryo was analyzed [35], showed that only 22% of embryos were uniformly diploid, 73% were mosaic and 5% had multiple non-mosaic abnormalities. Of those that were mosaic, nearly half had diploid/aneuploid mosaicism. Comparative genomic hybridization (CGH) is an alternative method of chromosome analysis originally developed to karyotype solid tumors. DNA from a test sample and control sample are labeled with either green or red fluorochromes. The DNAs are both hybridized in equal concentration to either metaphase chromosomes (mCGH) or to chromosome specific DNA probes fixed to a microscope slide (aCGH) [62]. The fluorescent intensity of the two fluorochromes is measured; if equal, a yellow color is obtained. A red or green color is seen if either the test or control DNA is present in excess. A commercial aCGH protocol (24Sure™ Bluegnome, Cambridge, UK) has been developed specifically for PGD, which contains about 3600 very robust BAC clones (average size of 150 kb) spaced at approximately 1 MB intervals with 25% genomic coverage and a resolution of 4–10 MB. The protocol can be completed in as little as 12 h, allowing a fresh Day 6 transfer after Day 5 trophectoderm biopsy. The results are presented as log_2_ ratios, as shown in Figure 2.

Early studies using mCGH of all blastomeres of non-transferred embryos [48,63] revealed that only 25% of the Day 3 embryos were totally euploid and that as high as two thirds demonstrated mosaicism. In one-half of these embryos, there were no normal cells (aneuploid/aneuploid mosaic), and 25% were diploid/aneuploid mosaics. These results led many to question whether the analysis of a single Day 3 blastomere was a good indicator of the developmental potential of the embryo.

Early data using aneuploidy testing with FISH in women of advanced reproductive age seemed to indicate a trend toward improvement in implantation and pregnancy rates [64,65,66,67,68]. These studies were mostly small, case-control studies in patients with advanced reproductive age or recurrent pregnancy loss. Despite somewhat encouraging results, statistical power could not be generated, and therefore, it was recommended that randomized clinical trials (RCT) be undertaken to prove that aneuploidy testing via PGD led to an increase in the birth of healthy children. Staessen *et al.* [31] in 2004 reported the first RCT comparing the results of blastocyst transfer with or without PGD using a FISH panel of seven chromosomes after the Day 3 cleavage stage biopsy of two cells. The results showed improved implantation and clinical pregnancy in the PGD group, but the difference was felt to not be of statistical significance. This group later performed a second RCT employing only single cell embryo biopsy, which produced improved results compared to the biopsy of two cells, but did not demonstrate any statistically significant benefit for the implantation or ongoing pregnancy in the PGD group, as compared to controls without PGD [33]. Overall, 10 RCTs have been performed to evaluate the efficacy of aneuploidy testing (for a summary table, see Fragouli and Wells [69]). The 2007 multicenter double-blind RCT performed by Mastenbroek *et al.* [70] reported that Day 3 blastomere PGD actually led to a decrease in the ongoing pregnancy rate, with the PGD group having a 25% ongoing pregnancy rate, as compared to 37% for the control group. This study garnered much media attention and caused much alarm among patients contemplating chromosome-based PGD. It was pointed out that the study was flawed on multiple levels, from patient selection, lack of embryo biopsy experience, selection of incorrect chromosomes for study and a high incidence of no results [71,72]. Some investigators pointed out technical issues with the RCTs already performed and felt that strict adherence to their protocol was necessary to show improved pregnancy rates with FISH [73].

At this point in time, there was a re-evaluation of the technology of the cleavage stage PGD for chromosomal aneuploidy testing. Relevant factors included: (1) The reduction in the implantation of embryos after blastomere biopsy; (2) The experience of the embryology laboratory with blastomere biopsy; (3) Proper cell fixation and cell lysis technique; (4) Hybridization issues, such as failure, split or overlapping signals, or nuclear damage with micronuclei formation; (5) The selection of optimal probes for FISH analysis (the use of seven probes for chromosome X, Y, 13, 16, 18, 21 and 22 was shown to account for 72% of chromosome abnormalities seen in spontaneous miscarriage [74]); and (6) The presence of chromosomal mosaicism, which leads to decreased accuracy and poorer pregnancy outcome [75,76].

The clinical utility of FISH-based PGD for chromosome abnormalities was seriously questioned, and it was recognized that in order to keep recommending PGD for patients with advanced reproductive age, recurrent miscarriage or multiple failed IVF cycles, a new RCT was needed using a different source of embryonic tissue for analysis [77,78]. In Europe, the analysis of polar bodies was selected, due to the legal restrictions on IVF and PGD present in many countries. Other groups turned their attention to the blastocyst and trophectoderm biopsy. At the same time, researchers in the field again considered CGH for chromosome analysis. As noted above, in 2000, mCGH was used by Wells and Delhanty [48] and Voullaire and colleagues [63] to analyze DNA obtained from multiple blastomeres of untransferred embryos after degenerate oligonucleotide primed (DOP) PCR. These studies indicated that 51% of cleavage stage embryos were aneuploid in every cell, and 24% contained a mixture of normal and abnormal cells, with only 25% being uniformly normal. Although superior in detecting chromosome anomalies compared to FISH with limited panels of chromosomes, mCGH is very labor and time intensive, thus requiring all embryos to be cryopreserved with a later frozen embryo transfer using embryos determined to be normal. Originally, this protocol was complicated by the fact that cryopreservation of biopsied embryos was very poor with low survival [79]. This improved dramatically with the introduction of embryo vitrification, with survival rates now over 90% and implantation similar to that of fresh embryo transfer [80].

Renewed interest in CGH resulted in this technology being validated for use in polar bodies [81,82,83], blastomeres and blastocysts [84,85,86]. Analytic improvement was accomplished with the evolution of mCGH to aCGH, where hybridization was performed on chips containing thousands of DNA segments fixed on a glass slide. Gutierrez-Mateo *et al.* [87] studied single blastomeres by aCGH after WGA in non-transferred embryos and compared the results to those obtained by FISH. They showed that aCGH detected 42% more chromosome defects, as compared to a panel of 12 FISH probes. Fragouli and colleagues [86] investigated the chromosomal status of blastocysts with mCGH. Some of these embryos had an abnormal Day 3 FISH result, which was confirmed by CGH, while the majority were donated, untested embryos, which were felt to represent what might be obtained in clinical practice. The aneuploidy rate of the blastocysts was 38.8%, which was lower than the 51% seen in Day 3 embryos [48,63]. Aneuploidy of all chromosomes was seen in blastocysts, including those of the larger chromosomes, as well as chaotic abnormalities (see Figure 2c). These findings dispelled previous beliefs that culturing to the blastocyst stage would effectively select against embryos with chromosome anomalies. The authors also showed the concordance of chromosome errors in the ICM and TE, indicating that preferential allocation of abnormal cells away from the ICM to the TE is not seen in humans.

In addition to mCGH and aCGH, single nucleotide polymorphism (SNP) arrays were validated to perform 24 chromosome analysis. SNPs are sequence variants that exist in the population, where at a particular locus, one of two (or more) nucleotides is commonly found. Biallelic SNPs, where one of two bases is seen, have proven to be useful in molecular cytogenetic analysis. The relative intensity of the two alleles, typically referred to as A and B, can be measured at heterozygous loci to detect duplications or deletions of chromosomal regions. SNP arrays also can reveal the parental origin of chromosomal errors by genotyping the parents. Rabinowitz and colleagues [56] have developed a bioinformatics algorithm using parental genotypes and reported on the chromosome number analysis of blastomeres from cleavage stage embryos. This showed increasing aneuploidy with maternal age originating from meiotic nondisjunction of the oocyte. SNP arrays also have the advantage of being able to detect polyploidy and uniparental disomy (UPD), which cannot be seen with CGH. Treff and colleagues [49] validated a high-density array with 262,000 SNPs using 99 WGA amplified single cells from chromosomally-defined lymphoblastoid lines. Three hundred thirty five WGA blastomeres were also studied with only 37.3% being euploid. This group also compared the performance of SNP arrays with that of interphase FISH by examining 160 blastomeres from arrested embryos and concluded that the SNP array was more accurate and FISH overestimated the aneuploidy rate [88]. Finally, CGH and SNP arrays demonstrated the significant mosaicism of cleavage stage embryos, which could lead to misdiagnosis and the non-transfer of potentially viable embryos.

Biopsy of embryos at the blastocyst stage was considered after the development of sequential media that allowed *in vitro* culture to Day 5 or 6 after fertilization. These embryos were felt to have higher developmental potential, and the testing of trophectoderm cells could lead to improved clinical outcome with less damage to the embryo. Fragouli and colleagues [85] studied 64 non-transferred blastocysts by mCGH, aCGH and a nine-probe FISH panel and showed that 42% of blastocysts were uniformly euploid, 30% uniformly aneuploidy, 17% were diploid-aneuploid mosaics and 15.4% composed of different aneuploid cells. FISH results were concordant with CGH in 10 of 12 embryos; however, in the remaining two, only the sex chromosomes were consistent. Northrup and colleagues [38] used SNP arrays to study 50 blastocysts and demonstrated that 62% of the blastocysts were uniformly euploid, 7.8% uniformly aneuploid and the remaining 30% mosaic, containing either diploid/aneuploid or aneuploid/aneuploid mosaicism.

Studies evaluating the ability of aCGH to detect mosaicism in blastocysts have been reported by Mamas *et al.* [89] and Novik *et al.* [90]. These studies created models of mosaic blastocysts by either mixing cells or DNA from euploid and aneuploid cell lines or embryos confirmed to be uniformly monosomic by FISH. The results showed that aCGH using the 24Sure protocol was able to clearly detect mosaicism when the proportion of abnormal cells was approximately 25%–30%.

As trophectoderm biopsy appeared to produce a more accurate assessment of the chromosomal status of the embryo, clinical studies were undertaken to evaluate the outcome of array-based PGD in infertile couples and those with recurrent pregnancy loss or repeated IVF failure. Schoolcraft and colleagues [91] studied 45 couples with infertility that underwent CGH-based PGD with trophectoderm biopsy and compared the results to 113 couples who had blastocyst transfer without PGD. Nearly 70% of the PGD couples achieved a clinical pregnancy as compared to 45% in couples where PGD was not performed. A clinical study in infertile couples using trophectoderm biopsy, SNP microarray analysis, embryo vitrification and subsequent frozen embryo transfer (FET) was reported by Schoolcraft and colleagues [92]. In the first 100 FETs, the biochemical pregnancy rate was 87% with a 73% clinical pregnancy rate (positive fetal heart rate) and a 2.3% miscarriage rate.

To avoid the need for embryo vitrification and later FET, Treff and colleagues [93] developed a new method for 24 chromosome analysis of blastocysts using real-time quantitative PCR (qPCR). This method used multiplex PCR in a 384-well plate format to amplify two areas on each arm of all chromosomes. The analysis can be completed in 4 h, allowing for fresh embryo transfer. Each of the methods for 24 chromosome analysis described above has their own specific advantages and drawbacks. An excellent summary comparing the different technologies has been published by Handyside [94].

The results of early and ongoing clinical trials of blastocyst biopsy and 24 chromosome analysis on improving implantation and ongoing pregnancy have been encouraging [95]. Harton *et al.* [96] have collected data from multiple IVF centers on patients undergoing IVF with PGD by aCGH after cleavage stage or trophectoderm biopsy. The pregnancy rates were higher for trophectoderm biopsy, but for both, the transfer of euploid embryos blunted the typical decrease in implantation and ongoing pregnancy associated with advancing maternal age. In other words, if euploid embryos were identified, their implantation potential was relatively independent of maternal age. This is what would be expected if aneuploidy was the major cause of implantation failure and miscarriage. Of course, as maternal age increased, so did the number of women who did not produce any euploid embryos. Scott *et al.* [97] have reported on an RCT involving 155 couples who were randomized to receive a Day 5 blastocyst transfer without PGD (control group) or a trophectoderm biopsy, comprehensive chromosomal screening by four-hour qPCR and Day 6 embryo transfer (study group). The rate of implantation and delivery was 66.4% with PGD and 47.9% in the control group. Delivery per cycle was 84.7% for the PGD group *versus* 67.5% in the control group.

These results also highlight one of the most important benefits of the increased implantation and lower miscarriage rate after 24 chromosome analysis; namely, the ability to optimize elective single embryos transfer (eSET). The goal is to reduce the incidence of twin gestation, which is associated with a 5–10-fold increase in the risk for fetal and maternal complications, including gestational diabetes, preeclampsia, premature delivery and low birth weight [98]. Since embryos that have a normal chromosome analysis have the highest developmental potential, it makes sense that the transfer of euploid blastocysts will optimize eSET. An RCT for eSET in good prognosis women <35 years of age was carried out by Yang *et al.* [99], who compared the transfer of a single embryo at the blastocyst stage on Day 6 that was either tested by aCGH or assessed by morphologic criteria only. In the aCGH group, the ongoing (>20 gestational weeks) pregnancy rate was 69.1% compared to 41.7% in the morphology only group. Another RCT was carried out by Forman *et al.* [100] in 205 women <43 years of age, but with normal ovarian reserve testing. The study group had chromosome testing by qPCR with subsequent eSET, while the control group transferred their two morphologically best embryos. The ongoing pregnancy rate per patient was similar (60.7% in the eSET group *versus* 65.1% after the two-embryo non-aCGH tested transfer); however, the multiple pregnancy rate was significantly higher in the two embryo transfer group (53.4%) *versus* the PGD eSET group (0%). The conclusion was that 24 chromosome screening after trophectoderm biopsy and eSET produced as high a pregnancy rate as the transfer of two untested embryos without encountering the increased risk for twin gestations.

### 4.3. Structural Chromosome Rearrangements

Structural chromosome rearrangements include reciprocal and Robertsonian translocations and inversions. These can be seen in approximately 1/500 live-born infants and 1/250 prenatal samples [101]. Individuals who carry balanced chromosome translocations (or inversions) generally have no clinical findings related to the translocation, but will produce high rates of abnormal gametes after meiotic segregation. The involved chromosomes will orient at the metaphase plate in a quadrivalent pattern and will segregate to the two daughter cells in one of 30 or so segregation patterns. These can contain areas of chromosome deletion or duplication, which can lead to pregnancy loss, failed implantation, apparent infertility or the birth of a child with physical and/or developmental disability.

Scriven and colleagues [102] published a method for the evaluation of segregation in translocations involving FISH using commercially available specific centromeric and sub-telomeric probes. Although this technique could identify embryos that were balanced for the involved chromosomes, it was subject to the same technical limitations described above for aneuploidy testing utilizing FISH. In addition, no information was provided for chromosomes not involved in the translocation, which could produce aneuploidy and decreased reproductive potential. Fiorentino *et al.* [103] developed a PCR-based method to evaluate translocations using STR markers that flanked the translocation breakpoints along with others to evaluate the copy number of other chromosomes. This was applied in 27 PGD cycles where 18 couples achieved a clinical pregnancy. Shortly thereafter, mCGH or aCGH was applied after WGA, and this analysis allowed not only aneuploidy evaluation of the translocated chromosomes, but also detected age-related aneuploidy in other chromosomes not involved in the translocation. In the report of Alfarawati *et al.* [104], where 16 couples with translocations underwent 20 cycles of IVF/PGD, 22% of embryos were chromosomally normal and 28.9% were balanced for the translocation, but had aneuploidy of other chromosomes. Fiorentino *et al.* [105] reported on 28 PGD cycles for translocation in 24 couples with aCGH and found that 16% of embryos were normal for all chromosomes, while 27.3% were normal for the translocation, but showed aneuploidy in other chromosomes. Treff *et al.* [106] validated an SNP array for the detection of translocations and evaluated 19 IVF/PGD cycles from 15 patients carrying a translocation. Of 122 normally developing blastocysts, 50.8% were normal or balanced for the translocation and 32% were euploid in the remaining chromosomes. Overall, including arrested embryos, 15.2% of all embryos produced were chromosomally normal. In 12 cases where the embryo transfer of normal embryos was carried out, a clinical pregnancy rate of 75% was seen. The implication of these results was that by using FISH for the PGD of translocations, approximately half of the embryos that were determined to be “normal” actually contained aneuploidy for other chromosomes not involved in the translocation and, therefore, not detected by the FISH analysis used in their case. Less than one quarter of the embryos produced in these cycles therefore were truly euploid and capable of producing a healthy pregnancy. The reproductive outcome in couples carrying a translocation is likely dependent on the shape of the quadrivalent and subsequent modes of segregation of the specific translocation and the risk of producing a viable abnormal gestation. Scriven *et al.* [107] argue that for fertile couples carrying translocations with a low risk of producing viable abnormal gestations, natural conception may be capable of producing a normal child in a shorter time and with lower cost than the use of IVF/PGD.

## 5. Next Generation Sequencing and PGD of the Future

Rapid advances in DNA sequencing technology have made it possible to generate very large amounts of sequence data with the use of high-throughput NGS and bioinformatics tools. The recent approval by the United States FDA of the Illumina MiSeq DNA sequencing platform is an indication that NGS will become integrated into multiple fields of medicine, including PGD. Typically, NGS protocols involve the fragmentation of the DNA to be sequenced into 100–200 base pair segments. Linker sequences, including molecular “barcodes” and oligonucleotides to attach the fragment to the flow cell, are ligated onto the fragmented DNA. On the flow cell surface, hundreds of thousands of the fragments are sequenced in parallel reactions involving the successive addition of fluorescent nucleotides and ultra-high resolution imaging of the successively added base. This approach is called “sequencing by synthesis”. The results are compared to a reference genome using a bioinformatics algorithm, and the protocol is repeated until a sufficient read depth is obtained by the sequencing of other fragments from the same genomic region. The use of barcodes allows multiple samples from different analyses to be sequenced simultaneously in the same flow cell, allowing efficiency of cost.

For PGD of single gene disorders using NGS, sequencing of the region containing the mutation until sufficient read depth is accomplished to be confident that the base calling is straightforward. Methods are now being developed to enrich WGA samples for specific genomic regions, where single gene mutations are present to increase the read depth of these sequences [108]. Treff and colleagues [109] evaluated NGS-based PGD for single gene disorders in six couples at-risk of transmitting either autosomal recessive disease (Walker Warburg syndrome, cystic fibrosis, familial dysautonomia), dominant disease (neurofibromatosis 1) or X-linked hypophosphatemic rickets to their children. NGS sequencing was carried out on excess embryonic DNA from trophectoderm biopsy using semiconductor sequencing technology with the Ion Torrent Personal Genome Machine (Life Technologies, Carlsbad, CA, USA). Aneuploidy testing was performed by qPCR. The genetic disease results were compared with Taqman allelic discrimination assays or PGD results from an outside reference laboratory. In all cases, the NGS results were perfectly consistent with those of the other two methodologies.

To determine chromosome copy number by NGS, the shotgun sequencing-chromosome mapping protocol originally developed by Fan *et al.* [110] for non-invasive diagnosis of cell-free fetal DNA in maternal circulation can be used. In this protocol, WGA embryo biopsy material is randomly fragmented, and the sequencing of 33–36 base pairs is carried out to allow the mapping of the fragment to the chromosome of origin. The number of fragments that map to a particular chromosome should be proportional to the copy number of that chromosome, with trisomic or monosomic chromosomes having more or less fragments, respectively. Since the DNA involved will contain only fetal sequence, as opposed to the situation in cell-free fetal DNA testing, where only 10% of the DNA is fetal in origin, relatively low average read depth and genomic coverage is adequate to accurately assess chromosome copy number. Yin and colleagues [111] studied trophectoderm biopsy samples from 38 donated blastocysts from 16 IVF cycles by both NGS and SNP microarray, with qPCR being used to define any inconsistencies between the two protocols. High throughput sequencing was performed using an Illumina HiSeq 2000 sequencer. An average of 9.3 million reads per embryo was obtained with an average 0.07× sequencing depth and 5.5% coverage of the whole genome. Results showed that 26 of the embryos (68.4%) were determined to be completely euploid, and 12 contained chromosome errors (31.6%). The euploid embryos were correctly identified by NGS and SNP array, and consistent abnormalities were identified in six uniformly aneuploid embryos. Segmental aneuploidy was detected by NGS and qPCR, but missed by the SNP array in two embryos, possibly due to bias from WGA. This group (Li *et al.* [112]) then used the NGS protocol clinically in 41 couples, analyzing trophectoderm biopsied WGA DNA, generating 8.2 million reads per embryo, with 5.5% genomic coverage. Uniformly euploid embryos were identified in 47.3% of biopsied blastocysts, and an ongoing pregnancy rate of 58.5% was recorded in 24 women who received a transfer.

Combining both aneuploidy testing and genetic disease diagnosis, Wells and colleagues [113] have reported the births of children to couples at-risk for cystic fibrosis or mitochondrial DNA defects after PGD. NGS using the Ion Torrent platform was performed on trophectoderm biopsy samples after MDA WGA to diagnose chromosome aneuploidy, as well as direct sequencing of the mutations present in the family. It was noted that the high throughput of the NGS system allowed simultaneous genetic analysis of up to 100 embryos, which could significantly reduce the cost of PGD to two-thirds of the current cost using aCGH. In addition, the pre-workup for single gene disorder PGD would no longer be needed, adding additional savings.

The above studies have demonstrated that NGS-based PGD can be accomplished accurately and with very high throughput. This will likely herald the move of PGD technology from PCR and aCGH to NGS analysis as the cost of sequencing continues to decline. A very significant question to be answered at this time is what will PGD look like in five to 10 years? As molecular technologies continue to evolve and allow us to accumulate huge amounts of sequence data, we need to decide what will be the role of PGD in the future. Some have suggested that PGD should be a part of all IVF cycles, especially where eSET is recommended. It is not clear, however, that universal PGD makes sense in terms of the extra cost for patients and the small number of qualified laboratories currently able to perform the procedure.

It has also been suggested that additional genetic screening be added to current PGD aneuploidy testing. In this regard, we will probably follow the experience of classical prenatal diagnosis, which is now, and will be in the future, most frequently performed by cell-free testing of fetal DNA in the maternal plasma. It has been discussed that several genomics providers will shortly introduce testing for chromosome microdeletion syndromes (DiGeorge/velocardiofacial syndrome, Williams syndrome, *etc.*) to their current aneuploidy screening protocol. It would be possible to do multiplex enrichment for these genomic abnormalities, which could be detected by NGS sequencing, likely in a protocol with embryo vitrification, data analysis and subsequent frozen embryo transfer.

The use of PGD for severe genetic disorders has always been non-controversial, and chromosome aneuploidy testing is only currently beginning to be more widely accepted as data accumulates from RCTs showing improved pregnancy and delivery rates. Other uses of PGD have been viewed more skeptically, such as HLA matching, adult onset genetic disorders, conditions with variable penetrance and non-medical sex selection. There is concern that PGD could be used for other non-medical conditions, such as height, hair color, memory or athletic ability, producing what has been called “designer babies”. As our understanding of genomic information improves, new prenatal tests will be developed, which are likely to be DNA sequence-based, and many could be adapted for prenatal or preimplantation diagnosis. It is not clear, however, whether PGD is the appropriate clinical setting to introduce new genetic testing strategies that have not yet been validated in the prenatal diagnostic clinic.

While the use of PGD to prevent the transmission of known serious genetic disease in a family is considered appropriate, the situation becomes less clear as more extensive genetic screening of embryos by technologies, such as whole exome or whole genome screening, become possible in the context of PGD. Questions arise regarding the moral obligation of the parents to perform extensive genetic testing on their embryos, simply because it is possible, as well as the obligation of health professionals to provide such testing.

Preliminary discussions regarding the future use of PGD and an ethical framework for its application have been carried out with panels of experts in the field [114,115,116]. Although there are few definitive conclusions from these meetings, the participants were unified in their belief that unless focused goals for the use of PGD are established, the procedure will encounter significant resistance for what will be perceived by some as eugenic pandering to parents’ fantasies about the “perfect” child [115]. In addition, extensive genomic sequencing will undoubtedly reveal unintended findings that may be clinically significant, or alternatively, variants of unknown clinical significance may be identified. This underscores the need for carefully obtaining the consent of couples for PGD along with both pre- and post-test genetic counseling to make sure the results are correctly interpreted and clearly explained.

## 6. Conclusions

PGD was initially performed nearly 25 years ago as an alternative for the prenatal diagnosis of single gene disorders in ongoing intrauterine gestations with the potential interruption of affected pregnancies. In the second phase of PGD development, cycles were performed for the detection of aneuploidy to improve the outcome of IVF in patients with translocations, advanced reproductive age, recurrent IVF failure or recurrent pregnancy loss. While there has been the general acceptance of PGD for genetic disorders, there has been skepticism of the benefit of PGD for chromosome aneuploidy, specifically the lack in multiple RCTs of a statistically significant improvement in the live birth rate with PGD, as compared to IVF without testing. With the analytic technology evolving from FISH-based testing to aCGH and SNP microarrays and, most recently, NGS, the quality of the results has improved. This has also been achieved with a change in the tissue biopsied from Day 3 blastomeres to Day 5 trophectoderm or polar bodies, which causes less harm to the embryo and, in the case of trophectoderm biopsy, provides a multi-cellular sample, which can mitigate the mosaicism common in cleavage stage embryos, as well as lower the risk of ADO. Initial reports have shown improved implantation and live birth rates with lower miscarriage rates in IVF when chromosomal PGD is performed. The identification and transfer of euploid embryos appears to blunt the effect of advancing maternal age and is the ideal method to select embryos for use in eSET to reduce the incidence of multiple gestations with IVF.

With the increasing use of NGS in PGD, both single gene disorder and chromosomal testing can be performed simultaneously on the same sequencing platform without the need for the pre-test workup of single gene disorders. In the near future, NGS is likely to be used to identify embryos with microdeletion syndromes or common pathologic copy number variations. New developments in sequencing technology and bioinformatics will likely allow even more sequence information to be rapidly generated from embryos. The objectives of such testing and the role that PGD should play in IVF will need to be further defined and validated, or the technology could be used for the identification of non-medical traits, such as stature, memory, hair and eye color or athletic ability. Without focused goals, PGD could be considered a mechanism to attempt the selection of the “perfect” child and will invoke the specter of “designer babies”.
